# Cancer testis antigen XAGE-1 is a promising marker for the diagnosis and treatment of ovarian cancer

**DOI:** 10.25122/jml-2021-0304

**Published:** 2021

**Authors:** Maysaa Ghazi Jumaa, Mukhallad Abdul-Kareem Ramadhan

**Affiliations:** 1. Department of Microbiology, College of Medicine, University of Maisan, Maisan, Iraq; 2. Department of Pathology, College of Medicine, University of Maisan, Maisan, Iraq

**Keywords:** XAGE1, ovarian cancer

## Abstract

Cancer testis antigens have been discovered in various cancers, and several studies have suggested that since they exhibit such distinct patterns of expression, these antigens might be attractive targets for cancer detection and immunotherapy. Our work attempted to clarify the function played by cancer-testis antigens in ovarian cancers, notably in the XAGE1 gene. The investigation was conducted on 74 tissue samples from newly diagnosed patients with ovarian cancer. The control group included twenty-eight benign ovarian tumors. The expression of XAGE1 mRNA was assessed using RT-PCR. Compared to benign tumors, cancer samples exhibited higher levels of XAGE1 gene expression, which was statistically significant (P0.01). There were no statistically significant differences between menopausal status and family history. Gene expression was substantially connected with age groups as the higher level of gene expression in patients 50–74 years of age (P 0.01) was seen. Mucinous tumors exhibited significant correlations (P0.01) across histopathological tumor types. In correlation with tumor stages, stage III was substantially linked compared to stage I (P0.01). In conclusion, we referred to the potential to use XAGE1 to discriminate malignant ovarian tumors as a diagnostic biomarker. The connection of high XAGE 1 level with advanced ovarian cancer stages has also been established, supporting XAGE 1’s proposed role in poor prognosis. Finally, finding the specific involvement of this gene in ovarian cancer and other kinds of malignancies may require further investigations.

## Introduction

Ovarian cancer has been considered one of the world’s most dangerous gynecological diseases, with high death rates in the last decade [1]. The frequency of new ovarian cancer instances has increased to 11.7 per 100,000 women per year and 7.4 per 100,000 women per year. This has led to a hypothesis that about 1.3 percent of women may be diagnosed with ovarian cancer at one point in their lives [2]. 30% of patients had advanced illness due to delayed diagnosis, recurrence of tumors, and resistance to chemotherapy [3]. Therefore, new goals and approaches were urgently needed to assist and promote the early diagnosis, prognosis, and development of new ovarian cancer therapy options. In ovarian cancer, a frequently seen genetic aberration and several driver mutations were discovered in ovarian carcinogenesis. However, the genome landscape in ovarian cancer was progressively disclosed with the genome-wide sequencing established in the last 10 years [4]. However, very few mutations are associated with cancer induction in these mutation-driver genes [5–6] because some of these mutations occur at low population frequencies or in a tiny fraction of individuals [7]. These genes, which are of particular importance due to their immunogenicity or limited modalities of expression, contain CTAs or cancer-testis (CT) [8–9]. CTAs are gene clusters of genes expressed solely in normal tissues, but large concentrations are observed in various types of tumor tissue. In germ cells, CTAs genes are also known as germline antigen genes. The CT genes are important in spermatogenesis and tumorigenesis, in addition to being immunotherapy targets. [9]. To date, 44 CT antigen genes or gene families have been discovered, including G antigen (GAGE), prostate-associated gene [PAGE], and melanoma-associated antigen (MAGEA) [10]. The XAGE gene is a member of the GAGE family, which includes multiple genes (GAGE-A, GAGE-B, PAGE, and XAGE) linked to apoptosis avoidance [11]. The XAGE 1 gene is found on the X chromosome; it is strongly expressed in germ cells and is commonly seen in malignancies such as breast cancer, rhabdomyosarcoma, germ cell tumor, and Ewing’s sarcoma [12]. XAGE 1 transcript variations include XAGE-1a, XAGE-1b, XAGE-1c, and XAGE-1d. These transcripts are expressed in various epithelial malignancies, including prostate, lung, and breast cancers, as well as Ewing sarcoma and metastatic melanoma [13]. The main transcript has been identified as XAGE-1b [14]. Previous research using SEREX analysis discovered that XAGE-1b might be involved in the production of antibody responses. CD4 and CD8 T cell responses against XAGE-1b in lung adenocarcinoma patients, leading to the hypothesis that XAGE-1b might be considered a potential antigen for tumor immunotherapy [15]. In this work, we looked at the expression of XAGE-1 in ovarian cancer tissues from Iraqi women to see if it had any diagnostic utility of XAGE-1 as a marker of ovarian cancer.

## Material and Methods

Following total abdominal hysterectomy and bilateral salpingo-oophorectomy (TAH-BSO), subtotal abdominal hysterectomy, vaginal hysterectomy, and endometrial biopsy, 74 Paraffin-embedded tissue blocks from patients with various stages of newly diagnosed invasive ovarian cancer were provided by certain Iraqi hospitals. Twenty-eight (28) samples of benign ovarian tumor tissues were used as a control. In addition, patients’ medical records were combed for information on their medical history and tumor characteristics. The paraffin-embedded tissue blocks were sectioned into 10m sections and placed in DNase-RNase tubes for molecular analysis. The samples were subjected to RNA extraction and molecular analysis using Reveres Transcription and Quantitative Real-Time PCR.

### RNA extraction

Total RNA was extracted from ovarian cancer and benign tumor tissues using the RNeasy FFPE Kit (Qiagen-USA), which is intended for purifying total RNA from FFPE tissue sections, according to the manufacturer’s procedure.

### Reverse transcription

Thermo-ScriptTM Reverse Transcription kit (Invitrogen/USA) was used to reverse transcribe the whole RNA. The cDNA synthesis was carried out in a 50 μl reaction volume. On the ice, all reaction mixtures were produced. 15 μl of RNA was denaturized in a 0.5 ml tube by incubation at 65°C for 5 minutes and then cooling on ice. The 5 x cDNA synthesis buffers were vortexed for 5 seconds before use. Table 1 shows the master mixture components and their volumes. Each reaction tube received the master reaction mix. After that, the samples were put in a 96-well thermal cycler and cycled under the following conditions: 10 minutes at 25°C, 10 minutes at 37°C, 60 minutes at 42°C, followed by 5 minutes at 75°C. The converted cDNA was stored at -80°C and then used as a template for PCR amplification. One of the housekeeping genes (PGK1) was used as a control gene.

**Table 1.** The reaction master mixture for cDNA preparation.

**Reagents**	**Volumes for 50 μl**
**Denaturized RNA**	15 μl
**Random hexameter primers 3μg/μl**	2.0 μl
**10 mM dNTP Mix**	5 μl
**5x cDNA synthesis buffer**	10 μl
**RNase OUT (40 U/μl)**	2.5 μl
**Thermo Script RT (15 units/μl)**	2.5 μl
**DEPC-treated water**	14.8 μl
**Total**	50 μl

### Constructs Synthesis

The target gene’s expression was confirmed using real-time quantitative qRT-PCR. To synthesize constructs for XAGE-1 and PGK1, qRT-PCR primers were developed to map the area of qRT-PCR amplification using cDNA from specific cancer patient samples with known expression for these genes, allowing us to manufacture constructs of the correct sizes.

### Real-time RT-PCR

Primers were generated using the primer 3 plus program, and target and endogenous genes were sequenced using the primers given in Table 2. The Applied Biosystems 7900 equipment was used to run quantitative real-time PCR tests in triplicate. For quantitative evaluation, the Real-time PCR system primer and SYBR Green master mix were employed. The amplification process was carried out in a 20 μl volume that contained 10 μl of SYBR Green master mix, 1 μl of primer mixes, 5 μl of RNase free water, and 4 μl of cDNA template. The Real-Time PCR methodology was as follows: stage 1: 50°C for 2 minutes, stage 2: 95°C for 10 minutes, stage 3: a two-step cycle process (95°C for 15 seconds and 65°C for 1 minute) repeated for 6 cycles, and stage 4 in a two-step cycle procedure (95°C for 15 sec. and annealing 61°C for 1 min) repeated for 40 cycles.

**Table 2.** Primers sequences of target and endogenous genes.

**Primers**	**Sequence**
**XAG1-CF**	5'-GGGCAGCAGACAGAAGAAGA-3'
**XAG1-CR**	5'-TTTGGTGAAAGCTGCAAAAC-3'
**PGK1-CF**	5'-GAGAAAGCCTGTGCCAACC-3'
**PGK1-CR**	5'-CTCCTACCATGGAGCTGTGG-3'
**XAGE1-F**	5'-GCGTCAAGGTGAAGATAATACCTAA-3'
**XAGE1-R**	5'-CATTTAAACTTGTGGTTGCTCTT-3'
**PGK1-F**	5'-GCGTCAAGGTGAAGATAATACCTAA-3'
**PGK1-R**	5'-CATTTAAACTTGTGGTTGCTCTT-3'

### The estimation of gene expression

The slope of a standard curve was used to calculate the efficiency of a real-time PCR reaction (E). The following formulae were used to calculate:



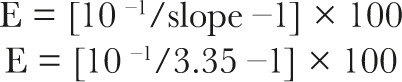



To compare across all samples, the Ct value is utilized. The Ct is proportional to the quantity of beginning mRNA of both the target gene (XAGE1) and the endogenous control gene (PGK1). The target gene’s relative fold change ratio in the sample was determined as follows:



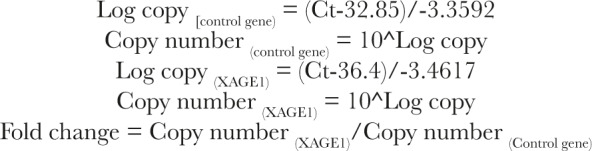



### Statistical Analysis

The Statistical Analysis System – SAS application was used to determine the influence of different variables in research parameters [16]. To compare means, the least significant difference – the LSD test (Analysis of Variation-ANOVA) or the T-test- was used in this research.

## Results

The patients’ ages ranged from 14 to 70 years, with a median of 47 years. According to the family history, all samples tested negative for an ovarian cancer family history. Table 3 lists the clinical characteristics of ovarian cancer samples. In terms of menopausal status, 34 (46%) of ovarian cancer patients were premenopausal, whereas 40 (54%) were postmenopausal. According to the International Federation of Gynecology and Obstetrics (FIGO) surgical staging method, the majority of samples (58.3%) were classified as stage I, while the remaining 16 (21.6%) were classified as stage III. According to the histological type, the samples were subdivided into serious tumors 32(43.24%), clear cell tumors 4(5.4%), clean tumors 4(5.4%), germ cell tumors 4(5.4%), and Burner 1(1,35%). In terms of malignancy status, ovarian cancer patients with XAGE1 positive gene expression had significantly higher levels of XAGE1 expression than benign tumor patients (LSD=2.319, p value=0.0001, P0.01). Table 4 shows the relationship between XAGE1 gene expression and clinic-pathologic characteristics. The present study shows no statistically significant differences in gene expression levels with menopause and family history, while significantly associated with age groups since higher levels of gene expression in patients aged between 50–74 years (LSD=1.762, p=0.0001, P 0.01) were observed. The statistically significant differences in the level of expression of the XAGE1 gene compared to the other types of tumors in association with histopathological types (LSD=3,821, p value=0.0001, P 0.01) were shown. In terms of tumor stages, levels of XAGE1 gene expression in stage III were substantially statistically different from levels of XAGE1 gene expression in stage I (LSD=1.948, p value=0.0001, P0.01).

**Table 3.** Patients’ clinical features.

**Age groups**	
Children (0–14 years)	2 (2.7%)
Teenagers and young adults (15–24 years)	1 (1.35%)
Adults (25–49 years)	28 (37.83%)
Old age (50–74 years)	43 (58.1%)
**Menopause**	
Premenopausal no. (%)	34 (46%)
Postmenopausal no. (%)	40 (54%)
**Family history**	
Positive no. (%)	0
Negative no. (%)	74 (100%)
**FIGO stages**	
Stage I no. (%)	58 (78.3%)
Stage III no. (%)	16 (21.6%)
**Histological type of tumor**	
Serous	32 (43.24%)
Mucinous	18 (24.32%)
Endometrioid	15 (20.27%)
Germ cell tumor	4 (5.4%)
Clear cell	4 (5.4%)
Burner tumor	1 (1.35%)

**Table 4.** Effect of clinicopathological features on XAGE1 gene expression in cancer patients.

XAGE1 gene expression	
**Tumor group**	Mean±SE of XAGE1 gene
**Benign tumors**	0.254±0.06
**Malignant tumors**	10.89±0.77
**LSD Value (P-value)**	2.319 **(0.0001]
** (P≤0.01).	
**Age groups**	Mean±SE of XAGE1 gene
**children aged 0–14 years**	0.0178±0.0115
**Teenagers and young adults aged 15–24 years**	0.00595±0.0022
**Adults aged 25–49 years**	0.8447±0.267
**Adults aged 50–74 years**	19.34±2.17
**LSD Value (P-value)**	1.762 **(0.0001)
** (P≤0.01)	
**Histological tumor type**	Mean±SE of XAGE1 gene
**Mucinous**	49.44±4.52
**Serous**	0.7252±0.284
**Endometrioid**	0.0566±0.032
**Germ cell tumor**	0.0178±0.84
**Burner tumor**	0.0134±0.0094
**Clear cell**	0.00229±0.0016
**LSD Value (P-value)**	3.821 **(00001)
** (P≤0.01)	
**Tumor stage**	Mean±SE of XAGE1 gene
**Stage 1**	0.01717±0.0085
**Stage 3**	57.06±3.06
**LSD Value (P-value)**	1.948 **(0.0001)
** (P≤0.01).	

## Discussion

The Cancer Genome Atlas (TCGA) has revealed driver mutations in ovarian carcinogenesis and novel techniques for targeted treatments [17]. However, only a few mutations in these mutation-driver genes may cause carcinogenesis [18–19]; among these genes, cancer-testis antigens (CTAs) or cancer-testis (CT) genes have piqued the interest of researchers due to their limited expression patterns and immunogenicity [20]. Utilizing the qRT-PCR method, we investigated the feasibility of using the XAGE1 gene as a diagnostic and prognostic marker based on gene expression levels in ovarian carcinoma tissues. The current study found that XAGE1-positive ovarian cancer patients had considerably greater levels of XAGE1 gene expression than benign tumor patients, indicating that the XAGE1 gene has a high specificity as a diagnostic marker for distinguishing malignant from benign ovarian cancer. Our findings were consistent with a recent study that found elevation of XAGE 1b mRNA expression in 64.4 percent of hepatocellular carcinoma tissue samples, but not in any benign liver tumors [21]. Gjerstorff *et al.* discovered significant levels of expression of numerous cancer-testis antigens (CTAs) in Tumorigenicity human mesenchymal stem cells, including XAGE-1 [22]. XAGE-1 expression profiles were comparable in lung cancer and immunogenic in patients [23]. Each of the CG genes (MAGEA1, NY-ESO1, and XAGE1) that James *et al.* assessed was not substantially expressed in benign prostate cell lines but was heterogeneously expressed in prostate cancer cell lines [24]. Sato *et al.* studied the prevalence of XAGE-1b mRNA among four alternative splicing variants, XAGE-1a, b, c, and d, in lung cancer patients [25]. The identification of XAGE1 expression positivity based on family history and menopausal status revealed no significant connection, but XAGE 1b mRNA was substantially higher in old patients aged 50–74 years than in other age groups. Previous research found that the expression of certain CT antigens was strongly related to the age, smoking history, and gender of lung cancer patients [26]. In 65.8 percent of patients with age −40 and <60, PAN *et al.* reported that elevated levels of XAGE 1b mRNA had been reported, whereas other research revealed no significant correlation of age with CTA expressions, including XAGE 1 in non-small cell lung cancers [27]. Nakagawa *et al.* demonstrated no statistically important connection with XAGE-1b mRNA expression between the patients of age or sex [23]. Regarding the histological tumor types, statistically significant variations in gene expression levels of XAGE1 have been reported in the mucinous ovarian tumors compared to the others. The CT genes, including XAGE1, have been calculated in several ovarian tumor subtypes and stage heterogeneity, which have been clearly limited, making statistical calculations problematic. These findings showed the need for additional investigations of the CT gene with various features of ovarian cancers. The statistically significant difference in expression levels in connection to stage III has been shown compared with stage I. Similar results were found in earlier research, including Pan *et al.*, who reported that XAGE 1b mRNA levels were raised in patients with hepatocellular carcinoma with higher stages of tumor node metastasis [II~IV][21]. Men *et al.* have discovered the pulmonary metastatic capacity of XAGE-1b in nude mice [28], but no association between CT expression and clinical variables like tumor history and histology has been observed in lung cancer patients [26]. Nakagawa *et al.* noted no association between the XAGE-1b mRNA expression or the lung cancer histologic level [23]. Our findings give further evidence of the potential involvement of XAGE 1 in invasive and metastatic cancer cells.

## Conclusion

In conclusion, the present work has shown considerable variations in gene expression in the use of the XAGE1 gene for the diagnostic biomarker to discriminate between non-malignant ovarian cancers. The combination of elevated XAGE 1 level with advanced-stage ovarian cancer supports the idea that the gene may be a poor prognosis predictor. Overall, since the studies of the gene XAGE 1 in ovarian cancer are very few, the clinical and pathological traits of our patients were difficult to compare with previous studies. Further studies are needed to detect the real role of this gene in ovarian cancer and to detect the gene’s immunogenicity and the possibility of using it as an immunotherapy target.

## Acknowledgments

### Conflict of interest

The authors declare that there is no conflict of interest.

### Ethics approval

Procedures in this study were approved by the Bioethical Committee of Medical College – University of Misan (No. 152 in 2021).

### Consent to participate

The consent of participation in the present study was taken from all patients from whom the samples were taken.

### Personal thanks

The authors would like to acknowledge the staff pathology department in the Medical College-University of Misan for their assistance and advice, and technical support. Many thanks to the patients who agreed to use part of their samples to support the present study.

### Authorship

MGJ developed the study conception, design, data analysis and draft manuscript preparation. MAKR performed the critical revision of the paper. MGJ supervised the research and complete funding acquisition. MGJ and MAKR gave final approval of the version to be published.
